# The complete mitochondrial genome of *Dictyostelium intermedium*

**DOI:** 10.1080/23802359.2021.1989332

**Published:** 2021-10-08

**Authors:** Kamonchat Prommarit, Passorn Wonnapinij

**Affiliations:** a Department of Genetics, Faculty of Science, Kasetsart University, Bangkok, Thailand; b Centre for Advanced Studies in Tropical Natural Resources, Kasetsart University, Bangkok, Thailand; c Omics Center for Agriculture, Bioresources, Food and Health, Kasetsart University (OmiKU), Bangkok, Thailand

**Keywords:** Mitogenome, Dictyostelids, Genome assembly, Public database

## Abstract

*Dictyostelium intermedium* is a member of dictyostelids, the unicellular eukaryotes with a unique life cycle, including a social cycle. Despite the high diversity of dictyostelids, only five species' complete mitochondrial genome sequences were reported. This study aimed to add the *D. intermedium* mitochondrial genome sequence to the list. The size of this genome is 58,627 bp, with 73.99% A/T, containing 62 genes located on one strand: 41 protein-coding genes, three ribosomal RNA genes, and 18 transfer RNA genes. The 41 protein-coding genes comprised 18 oxidative phosphorylation-related, 16 ribosomal, and seven hypothetical protein-coding genes. The *cox1/2* and *rnl* gene contained introns, similar to other species of *Dictyostelium*. The phylogenetic tree built based on 34 protein sequences supported the monophyletic clade of *Dictyostelium* and the dictyostelids' ancestor's position between the two dictyostelids orders: Dictyosteliales and Acytosteliales.

*Dictyostelium intermedium* sp. n. is a member of dictyostelids, the highly diverse group of unicellular eukaryotes with a unique life cycle consisting of a vegetative, sexual, and social cycle (Romeralo et al. 2012). It was firstly isolated from the forest humus and leaf mold, Peutjang Island, Java, Indonesia, and deposited at the American Type Culture Collection (ATCC) (https://www.atcc.org, email: tech@atcc.org) by Professor James C. Cavender (Cavender 1976). The nuclear gene phylogenies showed that it was clustered within the monophyletic clade of *Dictyostelium* (Schilde et al. 2019), one out of 12 recently classified genera (Sheikh et al. 2018). The nuclear protein-coding gene phylogeny further showed that *Dictyostelium* could be separated into five subclades and *D. intermedieum* is clustered with *D. discoideum* in one subclade (Schilde et al. 2019). Because only five species' complete mitochondrial genome (mitogenome) sequences were publicly available (Heidel and Glöckner 2008), this study aimed to add *D. intermedium* mitogenome to the list.

The 454 short-read whole-genome sequences of *D. intermedium* strain PJ-11 were retrieved from the ENA database under the accession SRR037009-17. These data were submitted by the Baylor College of Medicine Human Genome Sequencing Center (BCM-HGSC). The quality of short reads was evaluated by FastQC (Andrews 2010), then the reads were mapped to the genome of *Escherichia coli* (NC_000913) and *Klebsiella aerogenes* (NC_015663), the two bacteria commonly used for culturing dictyostelids, using BWA-MEM (Li 2013). The unmapped reads from SRR037010-11 and SRR037013-14 were assembled by Unicycler (Wick et al. 2017). The contig with the length between 40 and 60 kb was annotated. The mitochondrial protein-coding genes (PCGs) were identified by blastx (Camacho et al. 2009), MUSCLE (Edgar 2004), and ORFfinder (Souvorov et al. 2008). For ribosomal RNA (rRNA) genes, the small subunit rRNA gene (*rns*) was predicted by RNAmmer (Lagesen et al. 2007), while blastn (Camacho et al. 2009) and MUSCLE (Edgar 2004) were applied for identifying the intron-containing large subunit rRNA (*rnl*) and 5S rRNA gene. The databases for blastx and blastn were extracted from previously reported dictyostelids mitogenomes: *D. discoideum* (NC_000895), *D. citrinum* (DQ336395), *Heterostelium pallidum* (NC_006862), *H. album* (EU275726), and *Cavenderia fasciculata* (NC_010653). The nucleotide Ns were manually added to correct the reading frame of some protein-coding genes and then replaced by the highest frequency nucleotides found among short reads mapped to the N-containing regions in these genes by BWA-MEM (Li 2013). The tRNAscan-SE (Lowe and Chan 2016) with manual curation was applied to predict transfer RNA (tRNA) genes.

Amino acid sequences translated from mitochondrial PCGs were retrieved from six dictyostelids species and an outgroup (*Acanthamoeba castellanii*: NC_001637). Each gene was aligned using MUSCLE (Edgar 2004) and manually edited on Aliview (Larsson 2014), then concatenated by catfasta2phyml tool (Nylander 2011). The sequence matrix was applied to MEGA X (Kumar et al. 2018) for identifying the optimal evolutionary model. The maximum likelihood (ML) phylogeny was built by the RAxML (Silvestro and Michalak 2012) with 1000 bootstrap replicates.

The total length of *Dictyostelium intermedium* mitogenome is 58,627 bp (TPA: BK014289), comprising 45.30% A, 28.69% T, 17.02% G, and 8.99% C. This mitogenome contains 18 oxidative phosphorylation (OXPHOS) related PCGs, 16 ribosomal PCGs, seven hypothetical PCGs, three rRNA genes, and 18 tRNA genes. The *cox1/2* and *rnl* genes contained 3 and 2 introns, respectively. These genes are located on one strand. By revising the potential function of 11 hypothetical PCGs in the previously reported dictyostelids mitogenomes by blastx, 18 OXPHOS related PCGs, 18 ribosomal PCGs, and three rRNA genes have commonly been found. The paralogs of *rps3* and *rps11* gene could not be identified on the *D. intermedium* mitogenome; however, due to the conserved position across the previously reported five species, the two mitogenome regions located next to *rps3* and *rps11* gene possibly carrying these paralogs. The arrangement of non-hypothetical PCGs and all rRNA genes of *D. intermedium* is similar to that found in *D. discoideum* and *D. citrinum,* but not *D. purpureum* (Heidel and Glöckner 2008). Therefore, the mitochondrial genome of *D. intermedium* has size, content, and arrangement, similar to other species of *Dictyostelium*.

Amino acid sequences translated from 34 mitochondrial genes were retrieved from seven species, including *A. castellanii*. The maximum likelihood phylogeny was built based on the LG substitution model and a gamma correction for rate variation across sites with empirical frequencies (LG + G + F), as shown in Figure 1. This phylogeny presented two monophyletic clades: one contained only *Dictyostelium*, and another contained *Heterostelium* and *Cavanderia,* which corresponded to two orders: Dictyosteliales and Acytosteliales (Sheikh et al. 2018). The common ancestor of dictyostelids was placed between these two clades. The topology of this phylogenetic tree corresponded well with mitochondrial genome phylogeny (Heidel and Glöckner 2008), 18S rRNA gene phylogeny (Sheikh et al. 2018), and nuclear PCGs phylogeny (Schilde et al. 2019). Therefore, this phylogeny supported the monophyletic group of *Dictyostelium* and dictyostelids' ancestor's position between Dictyosteliales and Acytosteliales.

**Figure 1.**
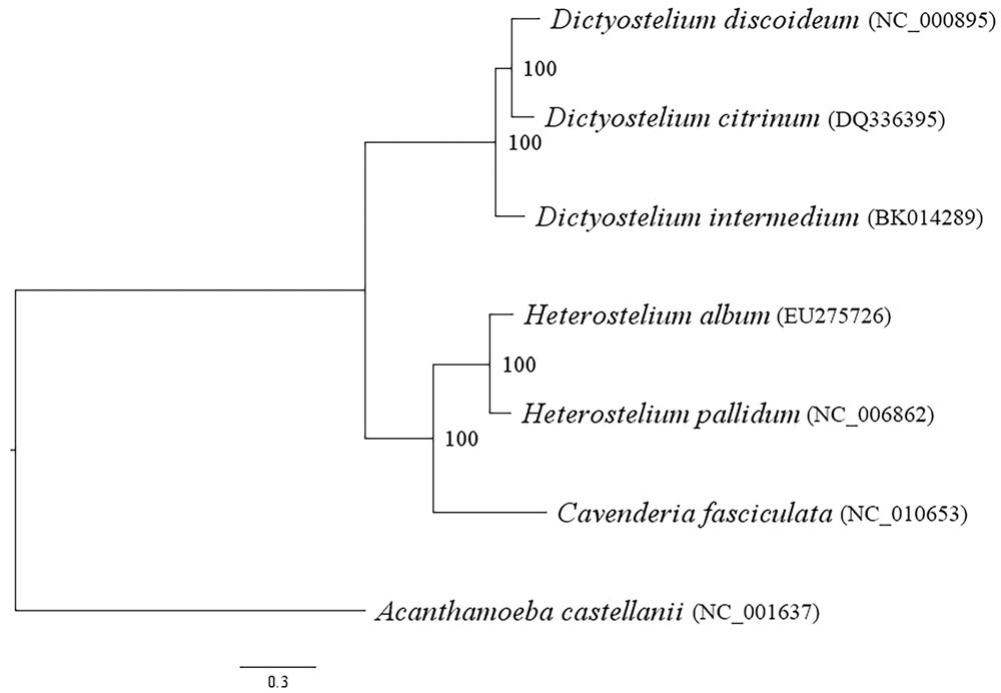
The maximum likelihood (ML) phylogeny built based on 34 proteins translated from mitochondrial genes of seven species of dictyostelids with LG + G + F model and 1000 bootstrap replicates.

## Supplementary Material

Supplemental Material

## Data Availability

The complete mitogenome sequence of *Dictyostelium intermedium* is available in the Third Party Annotation Section of the DDBJ/ENA/GenBank (https://www.ncbi.nlm.nih.gov) databases under the accession number TPA: BK014289. While waiting for the availability of the updated sequence record, the mapping results supporting the replacement of Ns in the sequence and the updated sequence are available upon request. This data was derived from the 454 short-read whole-genome sequence data of *D. intermedium* submitted by the Baylor College of Medicine Human Genome Sequencing Center (BCM-HGSC) and stored in the ENA database at https://www.ebi.ac.uk/ena/browser/home, accession number SRR037009-17.
