# Supplementary material for: The complete mitochondrial genome of Dictyostelium intermedium
Source: Mitochondrial DNA B Resour. 2021 Oct 8;6(11):3174–6. doi: 10.1080/23802359.2021.1989332 (PMC8567905; doi:10.1080/23802359.2021.1989332)
Supplement: Supplemental Material [file TMDN_A_1989332_SM8843.docx]

**Supplementary materials**


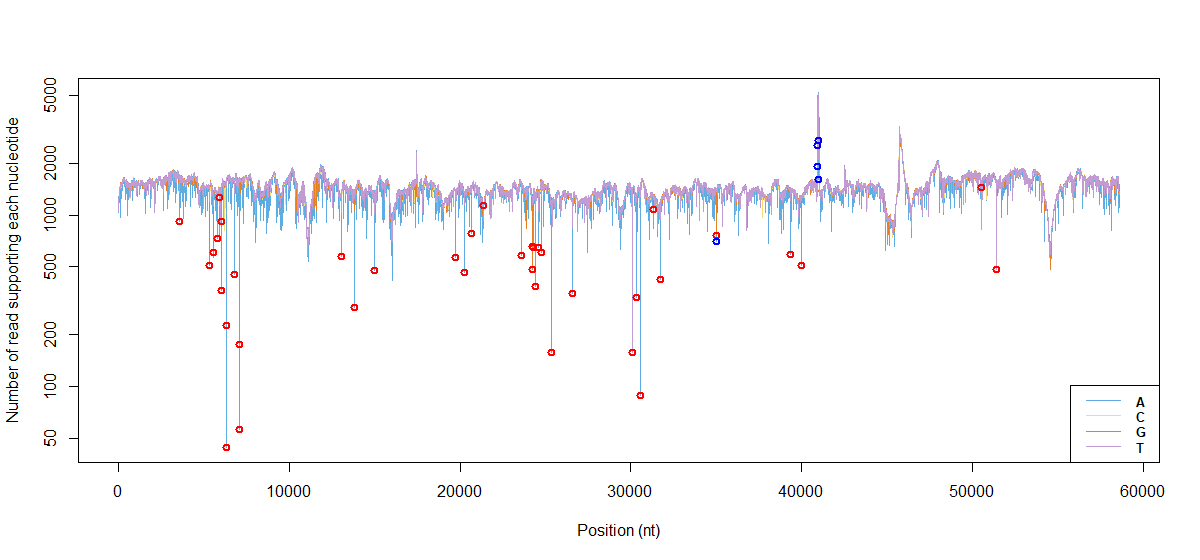


**Figure S1**: The number of short reads supporting the updated nucleotide sequence of the record TPA: BK014289 (updated_TPA-BK014289.fasta). The positions of Ns that were replaced by these reads were marked with red dots. The positions of other nucleotides that were revised based on this mapping result were marked as blue dots. The short reads applied to this mapping analysis were retrieved from the ENA database under the accession SRR037009-17.

**Table 1:** The information of the updated nucleotides regarding their number of short reads supports and locations on the mitochondrial genome of *Dictyostelium intermedium*.

| Position on the mitogenome | Gene or region in the mitogenome | Nucleotide in the current record | Nucleotide in the updated sequence | Number of read supports |
| --- | --- | --- | --- | --- |
| 3538 | *oMp21* | N | A | 911 |
| 5318 | *rpl2* | N | G | 510 |
| 5534 | *rpl2* | N | G | 607 |
| 5763 | *rps19* | N | A | 726 |
| 5888 | *rps3* | N | A | 1257 |
| 5996 | *rps3* | N | A | 911 |
| 5997 | *rps3* | N | A | 365 |
| 6295 | *rps3* | N | A | 228 |
| 6296 | *rps3* | N | A | 44 |
| 6775 | *rps3* | N | A | 453 |
| 7052 | *rps3* | N | A | 56 |
| 7053 | *rps3* | N | A | 175 |
| 13043 | *rpl16* | N | A | 572 |
| 13830 | *rpl5* | N | A | 289 |
| 15007 | *rps8* | N | A | 473 |
| 19722 | *nad11* | N | A | 569 |
| 20266 | *nad11* | N | A | 465 |
| 20679 | *nad11* | N | A | 781 |
| 21342 | *nad11* | N | A | 1019 |
| 23600 | *rps4* | N | A | 579 |
| 24215 | *rps4* | N | A | 652 |
| 24227 | *rps4* | N | G | 479 |
| 24278 | *rps4* | N | A | 651 |
| 24435 | *rps4* | N | A | 381 |
| 24598 | *rps2* | N | A | 649 |
| 24778 | *rps2* | N | A | 601 |
| 25346 | *rps2* | N | A | 159 |
| 26575 | *nad4* | N | A | 349 |
| 30065 | *cox3* | N | T | 159 |
| 30305 | *cox3* | N | A | 331 |
| 30545 | *cox3* | N | A | 89 |
| 31323 | *oMp05* | N | A | 1076 |
| 31720 | *oMp05* | N | A | 420 |
| 35039 | *cox1/2* | N | G | 756 |
| 35040 | *cox1/2* | G | A | 702 |
| 39375 | *atp1* | N | A | 587 |
| 40005 | *atp1* | N | A | 511 |
| 40926 | intergenic region | G | T | 1905 |
| 40957 | intergenic region | A | T | 2551 |
| 40984 | intergenic region | T | A | 1600 |
| 40997 | intergenic region | A | T | 2728 |
| 50550 | *atp8* | N | T | 1444 |
| 51424 | *nad3* | N | T | 481 |
